# Biophoton emission promotes the modern scientific utilization of medicinal plants: from theoretical integration to research applications

**DOI:** 10.3389/fchem.2025.1713717

**Published:** 2026-01-12

**Authors:** Baorui Cao, Zhiying Wang, Jinxin Du, Xue Li, Mingxia Wen, Huixin Yang, Xixue Lu, Meina Yang, Jinxiang Han

**Affiliations:** 1 College of Traditional Chinese Medicine, Shandong University of Traditional Chinese Medicine, Jinan, China; 2 Biomedical Sciences College and Shandong Medical Biotechnology Research Center, Shandong First Medical University and Shandong Academy of Medical Sciences, Jinan, China; 3 Institute of Biomedical Engineering, College of Life Sciences, Qingdao University, Qingdao, China; 4 Neck-Shoulder and Lumbocrural Pain Hospital of Shandong First Medical University, Shandong First Medical University and Shandong Academy of Medical Sciences, Jinan, China; 5 NHC Key Laboratory of Biotechnology Drugs, Shandong First Medical University and Shandong Academy of Medical Sciences, Jinan, China

**Keywords:** biophoton emission, Chinese herb, medicinal plant, holism, Chinese medicinal properties, quality control, real-time monitoring

## Abstract

Medicinal plants constitute the primary pharmacological source of Chinese medicine and their scientific interpretation is crucial for quality standardization and clinical guidance. Paradoxically, the contemporary evaluation model primarily adopts reductionism, inconsistent with the system-oriented holistic view of Traditional Chinese Medicine (TCM). In this review, the commonality between the organismic electromagnetic radiation field and the “qi” of TCM was deeply dissected from five levels. This reveals the fundamental consistency between the biophoton coherence and the holism of TCM. The scientific connotation of Chinese medicinal properties may be the ability and metric to regulate quantum superposition states of biological electromagnetic fields. Therefore, employing biophoton emission (BE)-based methods to investigate the medicinal properties of medicinal plants may provide scientific support that aligns more closely with holism. BE technology has shown great potential in quantifying medicinal characteristic parameters, including the quantization of Chinese medicinal properties, the identification of various processed products, and quality control. Especially in the real-time quality monitoring during the cultivation process of medicinal plants, BE characteristics can comprehensively reflect the natural and efficacious properties. This interdisciplinary thinking will help to establish a novel theoretical framework and methodological system to advance the modernization and development of medicinal plants.

## 1 Introduction

Traditional Chinese Medicine (TCM) emerged in the primordial societal era and evolved into a complete theoretical system during the Spring and Autumn and Warring States periods, with a history of approximately three millennia (Wang MM. et al., 2024). Grounded in the theoretical framework of Yin-Yang and the Five Elements doctrine, TCM posits that organic homeostasis is intrinsically linked to the dynamic balance of Yin-Yang. Consequently, its fundamental therapeutic principle involves managing disease by regulating this balance within the human body (L et al., 2025; Tang et al., 2016). With its ancient history, comprehensive and sophisticated theoretical system, and rich clinical experience, TCM has emerged as an integral component of the contemporary medicine system, garnering increasing recognition within the global medical community (Lin et al., 2022). The core concepts of TCM are the holistic view and syndrome differentiation (Wang et al., 2023). It possesses a rich array of therapeutic methods, including Chinese medicine, acupuncture, tuina, cupping, moxibustion, guasha, qigong, and dietary therapy (Ding et al., 2024; Wang et al., 2018). Among the therapeutic modalities in TCM, Chinese medicine holds a central and indispensable role. Compared to Western medicine, Chinese medicine has garnered increasing global scientific interest in recent years due to its advantages of fewer adverse effects and multiple targets (Liu et al., 2022).

Chinese medicine includes various pharmacological resources classified by taxonomic origin into four major categories: medicinal plants, medicinal fungi, medicinal animals, and medicinal mineral-based preparations. According to the Fourth National Census of Chinese Medicine Resources, the total documented species of Chinese medicinal resources amount to 18,817, with 15,321 species (81.4%) representing medicinal plants (Huang et al., 2024). This difference highlights the prominent position of plant-derived medicines. The “Chinese Materia Medica” documents approximately 9,000 herbal entries, exhibiting multifaceted pharmacological properties that serve as therapeutic cornerstones for diverse diseases in clinical practice (Yao et al., 2021). However, TCM has faced criticisms and challenges in its modernization and widespread application (L et al., 2025), particularly in the modern interpretation of TCM theories. As the most basic property of Chinese medicine, Chinese medicinal property theory has been severed in current research, and certain properties have even been completely neglected. A more significant problem is the lack of quantitative indicators for interpreting Chinese medicinal properties. Therefore, this greatly hinders the scientific and rational utilization and promotion of medicinal plants.

## 2 Basic theory of Chinese medicinal properties

The medicinal property refers to the nature, odor, and function of a drug. The concept of Chinese medicinal properties collectively describes the nature and function of Chinese medicines, which are directly related to their therapeutic effects. Specifically, it denotes the corresponding reaction that occurs within the human body after the administration of herbal decoctions, a unique property of herbal medicines. Its basic contents include the four qi (also known as the four properties), five flavors (sour, bitter, sweet, pungent, salty), meridian tropism, ascending, descending, floating, sinking (refer to the upward, downward, inward, and outward movement tendencies of herbal actions), toxicity and non-toxicity. Chinese medicinal property theory is the core theory of Chinese medicines, which is an important basis for guiding the clinical use and elucidating the mechanisms for treating diseases. The modern scientific interpretation of Chinese medicinal property theory is one of the key issues in the development and modernization of TCM, and it is also a necessary way for TCM to move from tradition to modernity (Song et al., 2023).

The narrow sense of medicinal properties mainly refers to the four properties of cold, hot, warm, and cool, also known as the cold and hot properties (Feng et al., 2020; Zhang X. et al., 2023), which is the core content of Chinese medicinal property theory (Xia et al., 2021). As the core theoretical framework of Chinese medicines, the concept of cold and hot properties provides a generalized classification of pharmacological actions. It describes the holistic bio-thermodynamic manifestations induced by herbal interventions in the human body. Cold-nature herbs relieve or eliminate heat-syndrome manifestations, including fever, polydipsia, irritability, constipation, erythema, and edema, by downregulating metabolic hyperactivity and inflammatory responses. They usually possess heat-clearing, fire-purging, blood-cooling, and detoxification pharmacological effects. Conversely, hot-nature herbs alleviate or eliminate cold-syndrome manifestations such as chills, cold limbs, lethargy, and diarrhea through enhanced energy metabolism and hemodynamics, exhibiting cold-dispersing, yang-fortifying, and fire-tonifying effects (Yang et al., 2025; Yu et al., 2020). The Five Flavors theory, an important component of TCM pharmacology, was first documented in Shennong’s Classic of the Materia Medica. This theoretical framework categorizes herbal flavors as pungent, sweet, sour, bitter, and salty. Initially derived from people’s perception and classification of herbal tastes and odors, it evolved into a functional property that reflects the regulation of physiological processes, representing the bioactive harmonization of Yin-Yang equilibrium and Qi-Blood (Zhang et al., 2025; Zhou et al., 2023). Pungent herbs promote sweating, dissipate cold, and eliminate evil energy; sweet herbs nourish deficiency, invigorate spleens, nourish body fluids, and replenish blood; salty herbs nourish yin, moisten dryness, diuretic, promote blood circulation, and resolve phlegm; sour herbs enhance defense function of body surface, stop sweating, and moisten lungs to stop coughing; bitter herbs purge intense heat, moisten the intestines, and promote defecation (Lin et al., 2025; Xu et al., 2025; Zhang and Li, 2018). Meridian tropism denotes the organ-selective biological distribution and site-specific actions of herbal compounds. This principle quantifies each herb’s affinity for target organs (Li et al., 2021): some herbs exhibit single-meridian specificity, while others demonstrate poly-meridian tropism. Renshen (the dried roots and rhizomes of *Panax ginseng* C.A. Mey.) Rhizome, for example, characterized by its sweet and slightly bitter flavors and warm nature, demonstrates multi-meridian tropism to the spleen, lung, heart, and kidney meridians. Its pharmacological activities include vital qi restoration and function enhancement of the spleens and lungs (Commission, 2020).

Current research on Chinese medicinal properties tends to focus on the four qi, especially for the distinction between cold and hot properties, which are mainly studied at the levels of energy metabolism (Tao et al., 2022; Zhan et al., 2022), material basis (Jia et al., 2017; Zhang CY. et al., 2023), and information (including omics, spectroscopic techniques, etc.) (Chen et al., 2018; Liang et al., 2023). For example, according to Li et al.’s report, Zhizi (the dried and ripe fruit of *Gardenia jasminoides* Ellis) and its processed products could significantly reduce the levels of substance and energy metabolism in rats, indicating that Zhizi showed the cold property (Li et al., 2024). Herbs of warming middle-jiao and dispelling cold, like Ganjiang (the dried rhizomes of *Zingiber officinale* Rosc.), Rougui (the dried bark of *Cinnamomum cassia* Presl), et al., show the warm nature by improving the thyroid function, and have an effect of warming and activating blood by promoting mesenteric microcirculation, thus to cure stomach excess-cold syndrome through relieving exuberant yin-cold and blood stagnation (Liu et al., 2014). Traditional studies on the five flavors were mostly based on oral tasting (Zhang, 2012). Still, nowadays, they are more based on the material basis (Ding et al., 2020; Sun et al., 2015), biosensor technology (Bu, 2021; Hao et al., 2023), and bionic technology of electronic tongue and electronic nose (Ren et al., 2023; Wang et al., 2022), which greatly solves the problem of excessive reliance on subjectivity and weak indicators. For example, Zhao et al. found that electronic tongue technology could reveal significant differences in the “taste” of Huangjing (the dried rhizome of *Polygonatum cyrtonema* Hua) from different origins (Zhao et al., 2019). This technology could also identify changes in “taste” resulting from different processing methods. Ding et al. conducted pharmacological studies on the chemical fractions of Zhike (the dried, immature fruits of *Citrus aurantium* L. and its cultivated variants), ultimately confirming that the sour taste is due to flavonoids, the pungent taste to volatile oils and alkaloids, and the bitter taste to polysaccharides (Ding et al., 2020). Tissue distribution (Xiu et al., 2013; Zhang et al., 2022), isotopic labeling (Teng et al., 2024), and systems biology (Liu et al., 2010; Teng et al., 2018) are commonly used to investigate the properties of meridian tropism. Studies on ascending, descending, floating, and sinking have been limited to literature reviews (Zhang MQ. et al., 2023) and pharmacological effects (Chen et al., 2016). Toxicity has been studied using methods such as serum medicinal chemistry (Huang, 2022; Zhang, 2020).

## 3 Quantum-based biophoton technology: an emerging platform for innovating Chinese herbal research

However, the above methods are mostly based on the actual effects of the drug to confirm its medicinal properties, while, particularly for plant-derived Chinese medicines, ignoring the formation and change patterns of medicinal properties during the growth process. Moreover, the therapeutic efficacy of Chinese medicinal plants originates from their intrinsic multi-component synergy and multi-target characteristics. However, prevailing reductionist approaches decompose holistic properties into isolated parts, which fundamentally conflict with TCM’s holistic view. In response to the current dilemma, an emerging technology - “biophoton” - is being introduced to address these issues. The inherently holistic nature of biophoton, reflecting systemic metabolism and carrying integrated physiological information, makes it a unique tool for the identification and assessment of Chinese herb, directly related to the holistic theory of TCM.

In 1905, A. Einstein published a revolutionary article called “The Light Quantum Hypothesis” (Shi, 2016) in which he first introduced the concept of “light quanta,” arguing that radiation is in the form of quanta in the process of emission and absorption and that radiation itself is also made up of light quanta. In 1916, A. Einstein published his paper “On the Quantum Theory of Radiation” (Einstein, 1916), proposing that electrons in an electromagnetic field undergo three processes: absorption, spontaneous emission, and stimulated emission. He believed that when electrons transition from a high-energy state to a low-energy state, some excess energy is released in the form of “light quanta.” In 1926, G. N. Lewis, an American physical chemist, first used the term “photon” instead of “light quantum” (Pais, 1982). The essence of photons is the quantized manifestation of electromagnetic radiation. Everything in the world, including biological organisms, exhibits inherent electromagnetic activity, or in other words, emits photons. This biologically generated photon is termed the biophoton, and the phenomenon of biological organisms emitting biophotons is called biophoton emission (BE).

Unlike high-intensity bioluminescence, which is specific to species such as krill, luminescent bacteria, and fireflies, BE is a much more general biological radiation phenomenon that can be found in various microorganisms, plants, animals, and even living isolated tissues or cells. Its radiation intensity is very low, with a typical intensity of only 100 counts/(s cm^2^), which is at least five orders of magnitude lower than the normal bio-fluorescence, hence it is also known as ultra-weak photon emission (UPE) or ultra-weak luminescence, and its spectral distribution is continuous in the range of 180–800 nm (Cifra and Pospísil, 2014). The first recorded instance of human exposure to BE dates back to the 1920s. While observing mitosis in onion root meristem cells, the Russian biologist Gurwitsch hypothesized that the cells under rapid division might emit imperceptible radiation, which was later validated as UPE (Uber, 1922). It is considered to be a product of life’s metabolic processes, arising from the transition of biomolecules from high-energy to low-energy states. This emission can reflect the complete information within a living system and is closely related to numerous life activities, including cell division and differentiation (Liu, 2013; Scholz et al., 1988), photosynthesis (Sun and Guo, 2023; Zhao et al., 2011), damage lesions (Gu and Biophotonics, 2012), and information transfer (Madl et al., 2017; Prasad et al., 2014). Furthermore, BE is highly sensitive to subtle changes in the external environment (Cifra and Pospísil, 2014; Popp, 2003).

Relevant studies have shown that BE originates from redox metabolic reactions within living cells (Bókkon et al., 2010; Pospísil et al., 2019), especially in the metabolism of reactive oxygen species (Jing et al., 2020; Mould et al., 2024). Redox reactions produce a large number of high-energy intermediates, such as peroxyl radicals, which decompose into electronically excited substances. However, biomolecules in the excited state are unstable and must be returned to a lower energy state. In the process of de-excitation of high-energy molecules, the excess energy is released in the form of photons (Prasad et al., 2018; Vahalová and Cifra, 2023). Considering that redox metabolic reactions, such as reactive oxygen species (ROS) metabolism, are ubiquitous biological reactions in living cells, the fact that BE is a ubiquitous life phenomenon in living organisms can be better understood. Subsequently, in 1988, Popp et al. (1988) proposed the coherence theory of BE, a quantum physical mechanism based on the interaction of radiation and matter, emphasizing that biophotons originate from a coherent non-linear interaction between multimode photon radiation in living matter and collective biomolecules (Gu and Biophotonics, 2012). Coherence theory suggests that biophotons originate from coherent fields (electromagnetic fields) within living tissues, that the electromagnetic fields of the subsystems are coupled to each other in a non-linear coherent relationship, and that the overall nature of the body’s electromagnetic field cannot be derived from a simple summation of the subsystems (Zhao XL. et al., 2013). Specifically, biologically active molecules generate ultraweak electromagnetic fields through quantum energy level transitions to emit BE. Crucially, these photon fields exist not in isolation but as coherent quantum states undergoing nonlinear superposition. The organismic electromagnetic field originates from constructive/destructive interference among sub-fields of all cells and tissues; this is the manifestation of quantum entanglement rather than linear summation. This thought regards BE as a holographic expression of systemic physiological states, conveying comprehensive information about the pathological and physiological conditions of the sample. Professor Gu (1988) argued that the living system is a non-linear, non-equilibrium, open system with a material basis for generating coherent radiation: activating substances (exciters formed by light or metabolic excitation of bases of DNA molecules), pump sources (various biochemical energies generated in metabolic activities) and resonant cavities (cells), and therefore the living system can generate coherent radiation. Biophotons based on the coherence theory are the product of the overall interaction of the living system, and can comprehensively reflect the full range of information within the organism, which seems to fit with the theory of the holistic view of TCM.

While both plant- and animal-derived biophotons exhibit ultra-weak characteristics, their origins diverge fundamentally. BE from animal systems arises predominantly from ROS metabolism within mitochondrial respiratory chains. Consequently, this low-intensity emission represents a typical manifestation of UPE. Through continuous investigation spanning over a decade, our research team has identified significant differences in UPE spectral and intensity characteristics of human fingertips across different physiological and pathological states (Yang et al., 2015). Distinct from animal systems, plants also exhibit chloroplast photon sources beyond mitochondrial ROS metabolism. Crucially, photosystem II reaction centers within thylakoid membranes generate singlet oxygen through charge recombination reactions, notably from interactions between ground-state molecular oxygen and excited triplet chlorophyll (Heta and Esa, 2022). Subsequent radical recombination releases photons in the visible spectrum, constituting a BE characteristic unique to plants. Notably, photosynthetic tissues, particularly leaves, exhibit delayed luminescence (DL), a unique BE phenomenon distinct from ultra-weak spontaneous photon emission (SPE). DL originates from the radiative relaxation of biomolecules sensitive to external stimuli such as light, primarily involving excited-state chlorophyll complexes within photosynthetic reaction centers. Characterized by non-exponential decay, DL fundamentally diverges from mono-exponential fluorescence decay.

## 4 Establishing the commensurability between quantum BE and TCM theory for feasible technology integration

From a philosophical perspective, the holistic view entails the harmonious unity of an entity’s various parts. In TCM, this view is primarily divided into three levels: human and nature, human and society, and the human body itself (Wang et al., 2019; Xian and Xiao, 2015). First, humans’ physiological and psychological activities and metabolic processes exhibit distinct seasonal, geographical, climatic environmental, and diurnal regulation patterns. The diversity in natural environments and organismic adaptability collectively shape distinct physiological and pathological organic states. Second, social stability, political systems, economic pressures, cultural patterns, and interpersonal dynamics inevitably modulate the physical and mental states of the human body. When psychosocial load exceeds individual buffering capacity, physical and mental dysfunction emerge. Third, and more importantly, the human body is an organic unity centered on the five zang-organs (heart, liver, spleen, lung, kidney). Through meridian pathways, these visceral hubs interconnect with the six fu-organs, sensory orifices, extremities, and osseous structures. Each functional unit works together to promote the body’s normal physiological activities. On the other hand, the mind dominates mental activities such as spirit, consciousness, and thought. Different emotions, meanwhile, are associated with specific internal organs. These organs carry emotions, which are the external manifestations of their physiological activities. Therefore, TCM diagnosis and medication should be guided by the holistic view. TCM treats diseases by using the whole herbal medicine, not just one or a few components. Therefore, the identification and evaluation of Chinese herbs cannot be limited to specific chemical components. Considering the extremely complex composition, it is quite difficult to identify Chinese herbs based on a thorough study of all the chemical components. Therefore, it is necessary to realize the overall evaluation of Chinese herbs when all the chemical components cannot yet be known. However, it is difficult to achieve this with the current physical and chemical methods alone. Western medicine, conversely, takes reductionism or divisionism as its scientific guiding principle, advocating the division of the whole into parts and then seeking the essence of the whole by combining the descriptions and explanations of the parts into one, which is contrary to the philosophical idea of the holistic view of TCM (Ding et al., 2023; Tang HA. et al., 2022). Therefore, the modern attempts to explain TCM in terms of Western reductionism are limited and unscientific, seriously hindering the evidence-based modernization of Chinese herbs.

“Qi” constitutes the core theoretical framework of Huangdi Neijing and TCM. It originates from the ancient Chinese “Qi Monism” philosophy, which posits “qi” as the fundamental substance constituting all things and thus explains the origin of the universe (Meng et al., 2017). All objective life phenomena, independent of human consciousness, including human physical organization and spiritual activities, are the result of the movement of “qi” (Ye et al., 2017). Although undetectable by scientific instrumentation, “qi” can be truly perceived through qigong practice and meditation of the ancients (Liu and Zhang, 2022), affirming its universality. Defined as a subtle and vital substance constantly fluxing within the human body (Zhang MY. et al., 2023), “qi” is similar to the concept of energy in modern biology, driving various life activities in organisms (Li and Ji, 2017). “Qi” ceaselessly circulates, propels, stores, and transforms in the form of energy to coordinate life processes. The theory of “qi” in TCM conveys the holistic idea that the whole is greater than the sum of its parts.

Physiological and pathological changes in any part of the body can disrupt the “qi” of the whole body. Conversely, the body’s physiological state can also be determined by its “qi”. When disturbances in “qi” cause pathological changes in a specific part of the body, the electromagnetic radiation state of the body also changes accordingly. This theoretical commensurability is strongly supported by empirical evidence. For instance, Yang et al. (2015) demonstrated that the SPE spectra from human fingertips could effectively discriminate between individuals diagnosed with “cold syndrome” and healthy subjects (Yang et al., 2017). This finding provides a direct biophysical correlate to the TCM holistic concept of “syndrome,” indicating that any systemic pathological state alters the organism’s global electromagnetic field, or “qi”. In other words, “qi” can be understood as an electromagnetic field. This inference has also been validated by multiple studies. Under natural conditions, infrared radiant tracks can be observed on the surface of the human body along meridian courses, and this phenomenon has been demonstrated to be universal across populations (Lan et al., 2017). Related research has confirmed that the human body in a qigong state can indeed emit microwaves, infrared radiation, and extremely faint visible light at specific wavelengths (Fan et al., 1990; Tian et al., 1995). These findings strongly support the biophysical hypothesis that the essence of “qi” is an electromagnetic field. Furthermore, studies examining DL kinetics or SPE intensity in samples administered Chinese herbal decoctions also reflected the holistic regulatory efficacy of Chinese herbs in BE parameters (Yang et al., 2022; Zhou et al., 2019). As mentioned earlier, the body’s metabolism is usually stimulated by warming herbs and slowed down by cold herbs. According to the theory of “metabolic luminescence,” these opposing effects on metabolism result in distinct radiative states within the body. These studies concretely demonstrate that BE characteristics can capture the holistic physiological status of an organism, thereby offering a modern scientific perspective through which to view the TCM concepts of “qi” and holistic regulation.

The holistic view in quantum physics posits that part-properties depend on the whole. Without the connection with the whole, the parts are meaningless (Xu et al., 2019). From a quantum-holistic perspective, “qi” and the electromagnetic fields underlying BE exhibit theoretical convergences. We envisage the two may have the same biological significance or material basis. Across five levels, including materiality, functionality, constancy, transformation, and intermediary (as shown in Table 1), Professor Han (Han, 2011a; Han and Han, 2010) systematically compared the characteristics of the electromagnetic field of body radiation and the “qi” of TCM. He proposed that the material basis of “qi” is the quantum of the electromagnetic radiation field and found the biophoton coherence theory consistent with TCM’s holistic view. From the perspective of quantum theory, the “qi” of TCM can be characterized by the electromagnetic radiation of the body, and the “syndrome” of TCM is the quantum superposition state of the electromagnetic radiation of the body (Pang and Han, 2012). We hypothesize that the state of “sufficient qi” described in TCM theory corresponds to a higher degree of coherence and moderate intensity in the BE field, whereas the state of “deficient qi” may manifest as reduced coherence and abnormal intensity. Based on this, it can be argued that the essence of Chinese herbs may be to regulate the quantum superposition state of the electromagnetic radiation of the organism to use the hot, cold, warm, and cool bias to adjust the bias of the human body and restore the organism to the most orderly quantum superposition state. Therefore, the scientific connotation of Chinese medicinal properties may lie in their ability to regulate the quantum superposition states of biological electromagnetic fields, serving as a metric for such regulation (Han, 2010; Han, 2011b). In other words, Chinese herbs restore the body’s biological photon field, known as “qi”, from an incoherent or disordered state associated with disease back to the coherent, orderly state characteristic of health. This process can be analogized to the evolution of the density matrix or the restoration of coherence in quantum systems. Due to the diverse internal structures and components of various Chinese medicinal plants, differences in their ability to adjust the superposition state of electromagnetic radiation in the body are inevitable. This ability may represent the medicinal properties or quality of these plants. However, this theory remains a scientific hypothesis and model, aiming to provide a possible materialistic explanation for the concept of “qi” in TCM theory based on electromagnetic radiation. It does not negate or replace traditional philosophical interpretations. Furthermore, BE represents only one aspect or manifestation of “qi”, not the concept in its entirety. The current model is better suited to explaining phenomena related to energy metabolism and holistic state regulation.

**TABLE 1:** Comparison of the characteristics of the organic electromagnetic radiation field and “qi” in TCM.

The five levels	Electromagnetic radiation field	Qi
Materiality	The smallest elementary particles that make up matter; wave-particle duality; measurable	The most subtle substance that constitutes the human body; invisible emptiness, tangible things; perceptible
Functionality	Inherent intrinsic properties of living phenomena; energy, momentum, and information are transferred through the exchange of light quanta	The basic substance that sustains the movement of life; “all diseases arise from qi”
Moveability	No static; light speed	Perpetual movement
Convertibility	E = m × c^2^	The exchange between mass (matter) and energy (function) and the transformation between tangible and intangible
Betweenness	Organ communication resonates with cosmic radiation and correlates with consciousness (the principle of immeasurability)	Man is interconnected with the universe, the earth, and the internal organs, meridians, and organs of the human body

**FIGURE 1:**
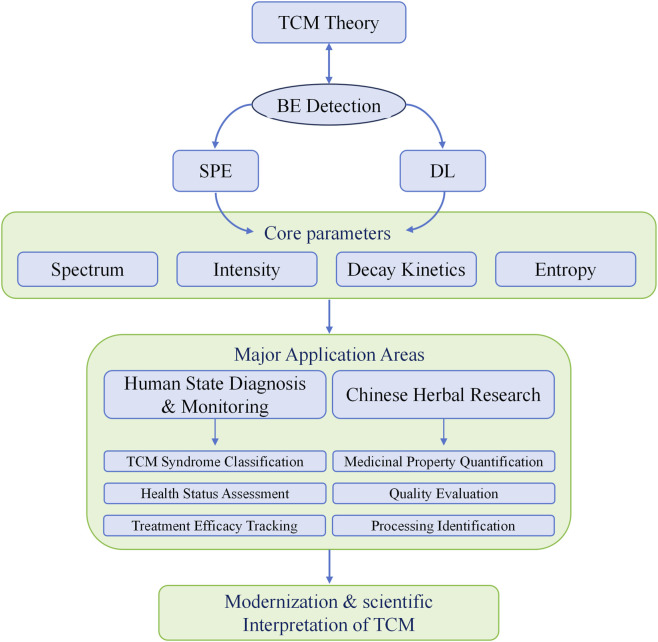
A framework for BE Technology in TCM research.

Nevertheless, utilizing BE detection technology to explore Chinese medicinal properties and identify quantitative indicators not only aligns with the holistic perspective of TCM but is also theoretically feasible. This approach provides a novel scientific framework for the modernization of TCM theory (Figure 1) and also has great implications for enhancing the safety and efficacy of herbal clinical applications. Specifically, the concepts of “qi”, “syndrome,” and “medicinal properties” in TCM theory are all highly holistic. The radiative characteristics of biophoton, including intensity, spectrum, and relaxation processes, can serve as comprehensive indicators to quantify these abstract, holistic concepts. This further illustrates the alignment between BE and TCM’s holistic perspective.

## 5 Biophoton technology in medicinal plant research: current methodological applications in specific fields and ongoing challenges

The application of biophoton detection technology to the field of medicinal plants was first reported in 1992 (Cui et al., 1992) when Cui Xiangyang et al. from the Air Force General Hospital used UPE detection technology to test and analyze four decoction pieces of medicinal plants: Ganjiang and Fuzi (the processed products of sub roots of *Aconitum carmichaelii* Debx.) are both hot medicinal plants possessing the effects of dispelling cold (Lei and Liu, 2021); Dahuang (the dried roots and rhizomes of *Rheum palmatum* L., *Rheum tanguticum* Maxim. ex Balf., and *Rheum officinale* Baill.) and Shigao (sulfate mineral gypsum group gypsum) are two cold medicinal plants that clearing heat and purging fire (Chen, 2019). The luminescence intensity of these four medicinal plants in the state of dried powder, aqueous decoction, and serum mixture showed an obvious increasing trend, and except for the luminescence value of Fuzi serum mixture, the luminescence intensities of Ganjiang and Fuzi were significantly higher than those of Dahuang and Shigao, indicating that the UPE detection technology has better feasibility and considerable application prospects in the research of Chinese medicinal properties. However, for more than 20 years thereafter, there was a virtual void of research content in the relevant areas, until Professor Han put forward the viewpoint that “the electromagnetic field of the body can characterize the qi of TCM,” the biophoton detection technology in the field of medicinal plants is rapidly carried out in related research.

### 5.1 Quantification of Chinese medicinal properties

#### 5.1.1 Meridian tropism and biophoton spectrum

Meridian tropism theory constitutes the cornerstone of Chinese medicinal property theory, precisely elucidating the body-target regions of Chinese herbs and providing critical clinical guidance for TCM practice. Following the conceptual advancement of BE coherence as a modern representation for, extensive research has emerged in the quantification of Chinese medicinal properties, initiating with pathbreaking investigations into DL excitation-frequency responses and meridian tropism. Experimental evidence indicates that the DL spectrum characteristics of dried herbal materials correlate with their meridian tropism classifications. Single-tropism herbal materials exhibit unimodal characteristics and dual-tropism herbal materials exhibit bimodal emission spectra. It can be reasonably assumed that multi-tropism herbal materials exhibit multiplexed spectral signatures. Critically, herbal materials that share the same meridian tropisms exhibit similar response peaks at comparable wavelengths. Specifically, Ma et al. (2013) found that 13 out of 14 herbs with single meridian tropism had single response peaks, and 12 out of 14 with double meridian tropism had double response peaks. This suggests that there may be a correspondence between the meridian tropism properties of medicinal plants and the response peaks of the DL spectrum. Wang (2014) found that there was a common peak in the wavelength range of 550–610 nm for single-meridian, double-meridian, and multiple-meridian herbs, which all attributed to the same lung meridian. Shi (2015) found that medicinal plants with identical flavors and meridians had very consistent intensities at the peaks, while those with the same properties of the four qi and five flavors but different meridians had different relative intensity values at each location. The peaks of Shegan (the dried rhizomes of *Belamcanda chinensis* (L.) DC.) and Qianhu (the dried roots of *Peucedanum praeruptorum* Dunn), which are attributed to the lung meridian, were at 550–610 nm, and the peaks of Shandougen (the dried roots and rhizomes of *Sophora tonkinensis* Gagnep.), which are attributed to both the lung and stomach meridians, were at both 550–610 nm and 610–665 nm. Except for DL, Wang (2016) also conducted a systematic analysis of SPE spectra across diverse herbal materials. This investigation revealed similar spectral characteristics of meridian tropism classifications to the above study. Dual-tropism herbal materials predominantly exhibited characteristic peaks within 715–870 nm, while single-tropism herbal materials within 610–715 nm. These findings verify that meridian tropism properties directly modulate the BE spectrum of dried herbal materials. Critically, they provide experimental validation for the hypothesis that meridian tropism fundamentally represents the frequency matching between organismic and herbal electromagnetic emissions.

#### 5.1.2 Chinese medicinal flavors and DL detection

The five flavors of medicinal plants, including sour, bitter, sweet, pungent, and salty, were first documented in the Huangdi Neijing and Shennong Bencao Jing. It initially denoted the true taste of oral medication, and is now encapsulated as systematic therapeutic actions through centuries of empirical improvement and clinical practice (Han et al., 2022). However, current flavor classification relies more on subjective sensory, lacking quantifiable identification criteria. Therefore, establishing more objective standards that align with TCM theory is more imperative. A systematic investigation of DL characteristics on 90 herbal materials of medicinal plants (Sun et al., 2017) demonstrated that bi-exponential fitting parameters of attenuation curves enable precise discrimination between sweet and bitter-pungent flavor medicinal plants. This DL-based classification was further validated through immunomodulatory tests: sweet-flavor groups significantly promoted IL-6 secretion, while bitter- and pungent-flavor groups significantly inhibited TNF-α and IL-6 levels. This implies that sweet-flavor medicinal plants are predominantly immunostimulatory, while bitter- and pungent-flavor medicinal plants are predominantly immunosuppressive. These findings establish DL spectroscopy as a sensitive analytical tool for medicinal flavor evaluation.

#### 5.1.3 Quantification of cold and hot medicinal properties

Cold and hot medicinal properties are thought to come from the body’s response to Chinese herbs. For example, Bohe (the dried aboveground parts of *Mentha haplocalyx* Briq.) causes the body to feel cold, while Ganjiang causes the body to feel hot. In the TCM theory, medicinal plants with cold properties are principally used to clear heat and toxins, and tonify yin essence. They are therefore clinically indicated for heat-excess syndromes such as sympathetic-adrenergic system hyperactivity and metabolic overactivation. Conversely, hot-property medicinal plants demonstrate warming and cold-dispersing capacities with yang-tonifying effects, therefore suitable for treating cold-deficiency disorders such as wind-cold invasion and neurological hypofunction (Liang et al., 2013). Despite their extensive clinical applications spanning millennia, the fundamental scientific basis underlying these cold and hot properties remains indistinct, resulting in significant challenges for quantitative characterization and modern interpretation.

Pang et al. (2016a) developed a novel approach to quantify cold and hot medicinal properties of medicinal plants. Utilizing a sensitive photomultiplier tube detection system, they measured DL of dried herbal materials and found that the mean intensity and statistical entropy were effective in distinguishing the cold and hot medicinal properties. This groundbreaking work was the first application of biophoton coherence for the holistic characterization of cold and hot medicinal properties, providing a non-destructive strategy to replace subjective and reductionist classification models. Furthermore, logistic regression modeling of DL parameters generated a discriminant function (Equation 1) that could accurately classify cold and hot medicinal properties with 79.4% overall accuracy (Pang, 2015). In another cluster analysis of DL parameters of 32 rhizome-originated and 24 root-originated dried herbal materials (Pang et al., 2016b), DL parameters can distinguish medicinal plant samples from different medicinal parts, as well as different cold and hot medicinal properties. This study also provides a new DL-based thought for the classification of Chinese herbs.

$$Y=\frac{1}{\left(1+e\times \left(-11.981+2.892\times S+0.270\times N\right)\right)}$$ (1)

S and N represent the statistical entropy and the mean intensity, respectively.

Subsequently, the bioindicator, *Tetradesmus obliquus*, has accelerated integrative research linking BE and Chinese medicinal properties. *Tetradesmus obliquus* is a ubiquitous freshwater microalga with the advantage of low difficulty in sterile cultivation and is widely used as experimental material for investigating water pollution and wastewater treatment (Xu et al., 2022). By combining BE detection technology with physiological characteristics, Nie (2016) determined the optimal growth stage and concentration of *T. obliquus*, as well as the optimal culture conditions (including light and temperature), for it to be used as an ideal bioindicator. Fu (2016) and Jia (2017) independently quantified DL dynamics of *Tetradesmus* obliquus exposed to decoctions of medicinal plants. The average intensity of DL (Equation 2) was calculated using the characteristic parameters A, B, and C fitted by the mathematical model of the “Gu parameter” (Equation 3). Subsequently, the linear equation (Equation 4) of the luminescence intensity with time “t” was obtained through linear fitting of I_w_ detected seven times in a row. Critically, the slope parameter K emerged as a highly sensitive biomarker for the hot and cold properties: the K values of the warm or hot herbal decoctions were significantly higher than those of the cold or cool herbal decoctions. This provides quantitative indicators for distinguishing the cold and hot medicinal properties of medicinal plants. Based on the DL detection and UPLC analysis, Min et al. (2023) found that the K value could also accurately differentiate the origin factor of Huangqin (the dried roots of *Scutellaria baicalensis* Georgi), indicating that the origin is an important factor affecting its medicinal properties. This provides new research ideas for the quantitative characterization of the medicinal properties of Huangqin and the identification of medicinal plants of different origins. According to one study (Yang et al., 2022), the cold and hot medicinal properties of more than 160 Chinese medicinal plants were successfully quantified using the DL assay of *T. obliquus*: warm and hot herbs have an increasing effect on the K value, whereas cold and cool herbs have a decreasing effect. This suggests that the DL detection method based on *T. obliquus* has a wide range of applicability and accuracy, and may serve as a “gold standard” for characterizing Chinese medicinal properties in the near future.

$$I_{w}=\frac{A\times B}{W}\times \left[\coth C-\coth\left(\frac{W}{B}+C\right)\right]$$ (2)

$$I_{t}=A\times {\mathrm{csch}\left(\frac{t}{B}+C\right)}^{2}$$ (3)

$$I_{W}=K\times t+b$$ (4)

I_w_ represents the average photon intensity of a DL detection, and w represents the overall measurement time. In the “Gu parameter” equation, A, B, and C are three parameters unrelated to time. A is an intensity parameter dependent on the sample’s nature, system structure, and illumination conditions; B is a characteristic time, related only to the nature of the sample itself; and C is a phase factor determining the initial state of the sample, which is sensitive to I_0_ of the DL.

In addition to direct assay of medicinal plants, researchers developed a pharmacodynamic-based approach to quantify medicinal properties through organismic BE characteristics. Zhou et al. (2019) measured SPE intensity ratios (abdomen/dorsum) of mice administered 20 herbal decoctions (10 hot herbs and 10 cold herbs) and correlated SPE data with hepatic biomarkers, including various antioxidant enzymes and ATPases. This investigation indicated that the SPE intensity ratios of mice in the control group were significantly higher than those in the cold-herb group, while significantly lower than those in the hot-herb group. This systemic strategy is firmly based on BE theory and aligns with TCM’s holistic framework. Within this framework, herbal bioactivity can be understood to manifest through biophysical means. Specifically, this photon emission results from transformations at the system’s micro-level.

We can reasonably propose the hypothesis that cold herbs may tend to reduce the intensity or frequency of BE associated with high metabolism and oxidative stress, whereas hot herbs may tend to elevate it associated with basal energy metabolism. By analyzing this correlation, we aim to find an objective characterization and modern interpretation based on biophysical signals (BE characteristic parameters) for cold and hot medicinal properties, which is holistic and functional.

### 5.2 DL detection and Chinese medicinal processing

Medicinal plants need to be processed before they can be used in clinical treatment, which is one of the characteristics of TCM. Chinese medicinal processing is a traditional method and technology of processing raw medicinal plants into medicinal products according to TCM theory based on the need for treatment with syndrome differentiation and the nature of the herbs themselves, using a variety of technical means such as stir-frying, steaming, dipping, and boiling. Chinese medicinal processing enhances the efficacy and reduces the toxic side effects (An et al., 2024). In today’s industrialized era, traditional and valuable Chinese medicinal processing technologies are gradually being lost or disappearing and being replaced by industrialized production modes, resulting in the quality of current Chinese herbs being severely degraded compared to traditional Chinese medicinal products (Wang and Zhou, 2021), making it difficult to meet the needs of clinical treatment. Therefore, it is imperative to improve the techniques of Chinese medicinal processing and to establish more comprehensive and effective methods for testing the quality of processed medicinal plants.

Sun et al. (2018) used a hyperbolic function model (Equations 5, 6) to extract four characteristic parameters (I_0_, Beta, Tau, and T) from the DL curves of dried herbal materials and found that the DL characteristics could reliably identify herbs such as Dihuang (the fresh or dried root tubers of *Rehmannia glutinosa* Libosch.) and Renshen and their processed products. Meanwhile, the DL characteristics of different processed products of the same medicinal plant were also significantly different. This non-destructive DL detection provides a promising novel technique for objective identification and quality analysis of Chinese medicinal processing products.

$$I_{t}=\frac{I_{0}}{{\left(1+\frac{t}{Tau}\right)}^{Beta}}$$ (5)

$$T=\left(e^{\frac{1}{Beta}}-1\right)$$ (6)

I_0_ is the initial intensity of the DL curve, Beta is an index factor associated with the rate of DL decay, Tau is a parameter that represents the DL characteristics, and T is the decay time.

Black ginseng and red ginseng represent two typical processed products of Renshen. Red ginseng is produced by single-cycle steaming and drying of fresh ginseng roots and rhizomes. In contrast, black ginseng is produced by multiple-cycle steaming and drying, resulting in a characteristic brownish-black appearance. Wang (2024) demonstrated that the DL decay kinetics of black ginseng powder progressively decayed with increasing processing cycles. The bi-exponential modeling (Equation 7) revealed consistent reductions in five key parameters: y_0_, A_1_, A_2_, t_1_, and t_2_. Concomitantly, common ginsenoside levels gradually decreased with increasing processing times, while rare saponins, like Rg3, Rg5, and Rk1, exhibited significant increases. Crucially, the seventh steamed cycle maximized immunomodulatory efficacy. In consistent findings, Yu (2024) demonstrated that red ginseng’s DL parameters and major ginsenoside content gradually decreased with extended steaming time. Conversely, rare ginsenosides and anti-inflammatory (neutrophil aggregation inhibition in zebrafish tails) levels exhibit significant increases. Collectively, these studies establish a relationship between processing methods and quality for Renshen, providing quantitative benchmarks for standardizing Renshen processing protocols.

$$y=y_{0}+A_{1}\times e^{-\frac{x}{t_{1}}}+A_{2}\times e^{-\frac{x}{t_{2}}}$$ (7)

y_0_ is the final value of photon emissions in the DL decay curve, A_1_ and A_2_ are the amplitudes of the exponential decay components, and t_1_ and t_2_ are time constants for the exponential decays.

Based on the DL assay of *T. obliquus*, Zhang (2018) conducted a quantitative exploration on the medicinal properties of processed products and compound formulations of Chinese herbs. Tiannanxing, derived from the dried tubers of *Arisaema erubescens* (Wall.) Schott, *Arisaema heterophyllum* Blume, or *Arisaema amurense* Maxim., is traditionally processed into two distinct medicinal forms: Zhitiannanxing and Dannanxing. Zhitiannanxing, obtained through thermal processing of raw Tiannanxing, is characterized by its warming property and is clinically utilized for dampness-drying and phlegm-resolving therapies. It has demonstrated therapeutic efficacy in wind-dispelling, anticonvulsant, and nodule-dissipating actions. In contrast, Dannanxing is produced by mixing the fine powder of Zhitiannanxing with animal bile (typically bovine, ovine, or porcine). It is primarily indicated for heat-clearing and phlegm-transforming interventions, with specific clinical applications on syndromes of calming wind, alleviating convulsions, and fright (Yang et al., 2020). Through comparative analysis, significant differences in the K-values of these two processed Tiannanxing derivatives were observed, with a strong positive correlation between K-values and β-sitosterol content. Notably, Chinese medicinal processed products, where medicinal properties remained consistent before and after processing, such as Gancao (dried roots or rhizomes of *Glycyrrhiza uralensis* Fisch., *Glycyrrhiza inflata* Bat., or *Glycyrrhiza glabra* L.) and its processed form Zhigancao (neutral), as well as Heshouwu (dried tubers of *Polygonum multiflorum* Thunb.) and Zhiheshouwu (slightly warm), exhibited no significant K-value variation. This consistency supports the validity of K-values as a quantitative indicator for characterizing the cold and hot properties. Furthermore, compound formulas categorized as warm or hot, including Sini Tang, Anti-Zuo Jin Wan, and Mahuang Tang, demonstrated significantly higher K-values compared to cold or cool formulations such as Zuo Jin Wan and Ma Xing Shi Gan Tang. These findings not only validate the K-value methodology in identifying Chinese medicinal properties but also provide a theoretical framework for investigating the thermodynamic properties of compound formulations in clinical practice. Jing et al. (2021) further demonstrated that the bioindicator system employing *T. obliquus* could effectively differentiate between processing- and origin-factors influencing the medicinal properties of Lianqiao (dried fruits of *Forsythia suspensa* (Thunb.) Vahl) and specification- and origin-factors affecting the medicinal properties of Sanqi (dried roots and rhizomes of *Panax notoginseng* (Burk.) F. H. Chen). This approach also quantified the K-value fluctuation ranges, providing quantitative benchmarks for medicinal assessment. These findings solidify biophoton detection technology as a critical tool in the discrimination of processed Chinese medicinal products, offering a non-invasive analytical system.

### 5.3 Biophoton technology and quality evaluation of medicinal plants

Ensuring stable and superior quality is fundamental to guaranteeing the clinical safety and therapeutic efficacy of medicinal plants (Li et al., 2022). Current quality evaluation focuses primarily on the level of bioactive components based on chromatographic pharmacodynamics (Zhang and Jiang, 2021). However, the chemical complexity makes it unrealistic to base quality testing on the level of all components (Lin et al., 2020). Existing other methodologies, including pharmacodynamics, serum medicinal chemistry, omics, and quality markers identification, although valuable (Ouyang et al., 2022; Ren et al., 2020; Tang YP. et al., 2022; Wang T. et al., 2024), either contradict the holistic TCM philosophy by isolating individual components or require invasive sample preparation, prolonged processing times, and high costs, potentially leading to irreversible structural damage and resource wastage.

In contrast, biophoton detection emerges as a non-destructive, highly sensitive technology that aligns with TCM principles. By capturing comprehensive physiological information encoded in photon emissions from biological systems, this technology reflects the dynamic energy-state interactions within biological samples. Its advantages include rapid data acquisition (only seconds to minutes, up to an hour, depending on the detection indicators), zero reagent consumption (no processing or extraction operation required, only requiring minimal consumption of water and electricity), and preservation of sample integrity, making it particularly suitable for quality evaluation of abundant medicinal plants. These characteristics position biophoton detection as a promising tool for bridging the gap between empirical TCM diagnosis and modern analytical standards.

The application of biophoton technology for quality evaluation of medicinal plants was first documented in 2016 by a collaborative study from Leiden University and the Meluna Research Institute, Netherlands (Sun et al., 2016a). This pioneering research employed DL analysis to compare unprocessed *versus* processed Chuanwu (dried mother roots of *A. carmichaelii* Debx.), wild *versus* cultivated Dahuang, and Renshen of varying growth ages. By performing bi-exponential fitting on the DL attenuation curve, the team identified five key parameters that exhibited statistically significant differences. Based on these findings, the researchers established a novel framework for quality evaluation through DL spectroscopy of dried herbal materials of medicinal plants, demonstrating its potential to distinguish processing methods, geographical origins, and growth stages with high resolution. This work also laid the foundation for integrating non-invasive BE techniques into standardized quality control systems of medicinal plants. Sun et al. (2016b) conducted a comprehensive study on Dahuang samples collected from varied altitudes in western China, investigating the influence of geographical altitude on medicinal quality through combining DL and bioactive component analysis. This study revealed significant differences in both DL parameters, including y_0_, A_1_, A_2_, and t_1_, and the contents of key bioactive components such as rhein, emodin, gallic acid, (+)-catechin, aloe-emodin glucoside, and sennoside A. Notably, strong correlations were also observed between DL parameters and component contents. These findings not only confirm the altitude-driven quality variation on Dahuang but also establish the practicality of DL detection as a rapid, non-destructive tool for origin and quality differentiation of medicinal plants. Both High-Performance Liquid Chromatography (HPLC) and DL assays have demonstrated responsiveness to pharmacological activity in Chinese medicinal extracts. For instance, DL parameters showed significant correlations with the anti-inflammatory efficacy of ginsenoside preparations (Sun et al., 2019a) and the laxative potency of Dahuang (Sun et al., 2020), suggesting a functional link between BE characteristics and bioactivities. Moreover, DL technology has also proven its identification capability in identifying the authenticity (genuine *versus* counterfeit samples (Sun et al., 2019b)) and harvest years (Sun et al., 2019a) of medicinal plants. These findings collectively validate DL assays based on dried herbal materials as a robust, rapid, and sensitive methodology for comprehensive quality evaluation of medicinal plants.

### 5.4 Related research in other fields

Zhao XJ. et al. (2013) investigated the effect of 60Co-γ irradiation on the UPE intensity of Chinese herbal granules. The results revealed a statistically significant increase in UPE intensity of pediatric diarrhea Lixian granules and Banlangen (the dried roots of *Isatis indigotica* Fort.) granules after irradiation, demonstrating the high sensitivity of UPE technology to detect irradiation history in Chinese medicinal granule preparation. *Isatis indigotica* Fort., a plant with medicinal and edible properties, is utilized in TCM as Daqingye (dried leaves) and Banlangen (dried roots). Pang et al. (2017) found a continuous decline in both SPE intensity and initial values of DL (I_0_) as germination rates increased, indicating a direct correlation between seed germination and BE characteristics. This discovery not only validates the feasibility of using biophoton technology for seed viability assessment but also provides a preliminary foundation for herbal seed quality control and agricultural breeding.

### 5.5 Current challenges and future development prospects

The integration of BE technology into TCM research provides a paradigm-shifting approach. However, to transition from a promising concept to any practical application, several conceptual and methodological challenges must be addressed.

#### 5.5.1 Methodological reductionism in BE-based property assessment

A primary challenge lies in the prevailing methodology. While BE is inherently holistic, current research often deconstructs Chinese medicinal properties, such as the four qi, five flavors, and meridian tropism, into isolated variables for independent correlation with BE parameters. This reductionist thought contradicts the foundational TCM principle that these properties are interconnected and manifest as an integrated systemic whole. Future studies must develop experimental designs and analytical models to capture the synergistic nature of medicinal properties. It aims to treat the BE characteristic as a holistic reflection of the herbal total bioactivity rather than a collection of discrete indicators.

#### 5.5.2 The limitation of dried herbal materials

Another critical limitation is the inappropriate application to dried herbal materials of medicinal plants. While biophotons are widely believed as endogenous photon emissions generated from metabolic redox reactions in living organisms, the direct measurement of dried herbal materials is unable to capture these biologically relevant metabolic signals. This discrepancy highlights a fundamental methodological error: current BE-based studies often overlook the temporal and spatial specificity of photon generation within the organism, which is crucial for accurately reflecting holistic pharmacodynamic mechanisms of medicinal plants. Future work should prioritize the analysis of fresh plant tissues, living cell cultures, or *in vivo* models to obtain physiologically relevant BE data.

#### 5.5.3 Integrating cultivation into the quality control system

Furthermore, the cultivation process of medicinal plants remains outside the quality control system, creating a critical loophole in the herbal supply chain. During the growth cycle, medicinal plants are susceptible to multiple factors, including germplasm diversity, climatic variability, and environmental stressors (both biotic and abiotic), which collectively hinder the secondary metabolite biosynthesis and herbal quality formation. The existing quality control system further exacerbates this issue by adopting a passive, post-harvest evaluation model. This model merely distinguishes “good” from “bad” herbs without addressing the fundamental cause of quality variation. In fact, BE characteristics from medicinal plants during their growth process serve as a rich source of information reflecting vital life activities such as growth, cellular metabolism, and the biosynthesis of secondary metabolites, all of which are critical indicators of plant physiological status and herbal quality. Therefore, making BE dynamic monitoring during the cultivation process of medicinal plants, the core link of quality monitoring, can help break the current dilemma.

#### 5.5.4 Feasibility and future directions of monitoring the growth process of medicinal plants based on BE

The intrinsic link between BE and core physiological processes highlights the practical feasibility of this approach. As a product of redox metabolism and an indicator of plant system status, BE serves as a real-time “sentinel” for plant health and metabolic activity. Therefore, capturing the BE’s dynamic changes during the cultivation of medicinal plants helps to investigate its circadian rhythms and dynamic responses to biotic/abiotic stressors (e.g., drought, salt, and pathogens), and cultivation practices. Simultaneously, to fully decipher BE variation patterns and establish causal relationships, it is essential to systematically integrate BE with metabolomics, transcriptomics, and proteomics. BE signatures provide holistic real-time predictions of plant physiological states, while multi-omics analysis fully reveals the underlying molecular mechanisms. Concurrently, machine learning algorithms (e.g., random forests and support vector machines) can be used to train models to directly predict key quality indicators from rapid, non-destructive BE measurements, such as concentrations of key bioactive compounds or specific pharmacological activities. By systematically analyzing how BE characteristics evolve throughout the entire growth cycle, it will become possible to develop a novel methodology for real-time and non-invasive monitoring of both the biochemical composition and quality formation of medicinal plants. Such an approach has enormous potential to address the current challenges faced by medicinal plants by enabling active interventions based on real-time biological feedback. Ultimately, integrating BE-based monitoring methodology into the cultivation process would significantly promote the scientific and technological modernization of TCM.

## 6 The dual-properties of Chinese medicinal properties and quality monitoring in the cultivation process

### 6.1 Neglected natural properties - equally crucial for Chinese herbs

Ancient Chinese doctors generalized the diverse functions and effects of medicinal plants in treating diseases, summing up the concepts of four properties, five flavors, meridian tropism, ascending, descending, floating, sinking, as well as toxicity and non-toxicity, which are Chinese medicinal properties. While these concepts are undoubtedly correct and have proven effective in clinical practice, comparatively speaking, a critical limitation lies in their primary focus on the interactions between herbs and organisms, emphasizing the manifestation of therapeutic effects within the human body. This approach, however, neglects the intrinsic properties of medicinal plants arising from their interaction with the natural environment during the growth process. Therefore, this bias results in an incomplete characterization of herbal properties and quality.

According to Academician Tang et al. (2010), Chinese medicinal properties are the drug properties that are highly summarized using Chinese philosophical methods, which are formed in the process of adapting to the changes of natural environmental factors, and it is an important basis for the clinical symptomatic use of drugs and the regulation of patients’ conditions, including its natural properties and efficacious properties. We usually talk about Chinese medicinal properties, that is, the four qi, five flavors, meridian tropism, and other contents, collectively referred to as the efficacious properties. According to the changes in yin-yang and cold-hot in the human body after medication, Chinese herbs can be divided into cold, hot, warm, and cool. Based on the authentic taste and the effect, Chinese herbs can be divided into sour, bitter, sweet, pungent, and salty. When a Chinese herb enters the body, it can be classified under the corresponding meridian according to where it works, e.g., if it works in the lungs, it will be classified under the lung meridian. These properties are all distinguished from the perspective of the interaction between the drug and the organism. Natural properties refer to the intrinsic properties of medicinal plants arising from their interaction with natural environmental factors during the growth process, including shape, color, texture, odor, and chemical composition, which is the material basis of the efficacious properties of Chinese herbs. However, the BE characteristics of medicinal plants during their growth cycle, which may convey important information about their properties, are currently being overlooked. This greatly limits the integrated development of biophoton technology and the modernization of Chinese herbs.

### 6.2 Systematic exploration of BE characteristics of medicinal plants during the growth process

Existing literature on BE in medicinal plants has primarily focused on post-harvest processed materials, including dried herbal powder and decoction. While these approaches provide herbal BE characteristics, they fundamentally lack the real-time quality monitoring during cultivation, which is essential for capturing the dynamic physiological responses of medicinal plants to environmental stimuli during the growth process. To address this critical methodology gap, exploratory investigations on the BE dynamics of fresh medicinal plant samples throughout the entire growth and developmental cycle have been conducted. This methodology enables the direct observation of environmentally induced BE signals, offering a novel perspective on the intrinsic physiological and pharmacological properties of medicinal plants. By correlating BE data with secondary metabolite biosynthesis, stress response, and omics markers, this approach aims to establish a quantitative link between BE parameters and herbal quality.

Zhao (2014) conducted a systematic investigation into the methodological establishment and optimization of BE detection for fresh medicinal plants. Using Jinyinhua (flower buds or early-blooming flowers of *Lonicera japonica* Thunb.) as a model, this study systematically explored three BE characteristic parameters, including initial intensity (I_0_), coherence time (T) derived from Gu parameters model (Equations 8, 9) and count per second (CPS) of SPE. The results demonstrated that these three indicators exhibited statistically significant discriminative capacity across three dimensions: growth developmental stages, health status, and various varieties. This methodological breakthrough provides a non-destructive, quantitative model for real-time monitoring of herbal quality during cultivation, addressing the critical limitation of post-harvest BE detection in capturing dynamic growth processes. A subsequent study (Zhao et al., 2017) demonstrated variety and growth stage specificity in the bioactive components of Danshen (dried roots and rhizomes of *Salvia miltiorrhiza* Bge.) and Jiegeng (dried roots of *Platycodon grandiflorum* (Jacq.) A.DC.), with statistically significant differences observed in key components across different varieties and growth cycles. Notably, these biochemical differences were quantitatively verified in the SPE detection, where characteristic parameter - CPS showed strong correlations with the secondary metabolite levels. This finding establishes an intrinsic quantitative connection between the key BE parameter and herbal quality, validating the value of BE detection as a non-destructive and quantitative quality evaluation tool for medicinal plants. Cao et al. (2023) established a multidimensional validation framework that integrates BE characteristic parameters, bioactive components, and physiological indices. Using Honghua (dried flowers of *Carthamus tinctorius* L.) and Yimucao (fresh or dried aerial parts of *Leonurus japonicus* Houtt.) as models, this study demonstrated that key BE parameters, namely, I_0_ and CPS, exhibited strong positive correlations with bioactive component levels and photosynthetic pigment contents.

$$I_{0}={A\mathrm{csch}}^{2}C$$ (8)

$$T=B\left\{\ln\left(\sqrt{m}\sinh C+\sqrt{\left(m\sinh^{2}C+1\right)}\right)-C\right\}$$ (9)

The value of the factor “m” ranges from 2 to 4, with m typically being three in related studies.

Current investigations into the relationship between herbal quality and abiotic stressors have adopted an integrated experimental framework, combining drought stress, salinity stress, and heavy metal stress treatments with multi-omics analysis to elucidate the molecular mechanisms underlying stress-induced herbal quality reduction. This approach correlates BE characteristics of medicinal plants with physiological indices, bioactive component contents, and pharmacological activities, establishing a multidimensional diagnostic network for herbal quality evaluation. Wang et al. (2025a) pioneered this integrative methodology by establishing stress models on *I. indigotica* Fort., a cruciferous medicinal plant, under salinity stress and drought stress conditions. This study demonstrated that key BE parameters, I_0_ and CPS, exhibited statistically significant correlations with stress-responsive physiological indices (including photosynthetic pigment content, relative electrical conductivity, and reactive oxygen species level) and bioactive component contents in Daqingye. Meanwhile, joint transcriptomic and proteomic analyses revealed that changes in BE characteristics and herbal quality under stress conditions correlated with the downregulation of genes involved in photosynthesis and energy metabolism (e.g., *HemB*, *PsbB*, and *RBS2*), as well as indole alkaloid biosynthesis pathways (e.g., *TRP3* and *TRPA*) (Wang et al., 2025b). The collective findings from these studies confirm that BE assays of medicinal plants hold valuable potential in the quality control system of Chinese herbs. By non-destructive and real-time monitoring of physiological and biochemical dynamic during cultivation, biophoton technology bridges the gap between single efficacious properties and holistic properties, ultimately enhancing the standardization and predictability of herbal plant production.

**FIGURE 2:**
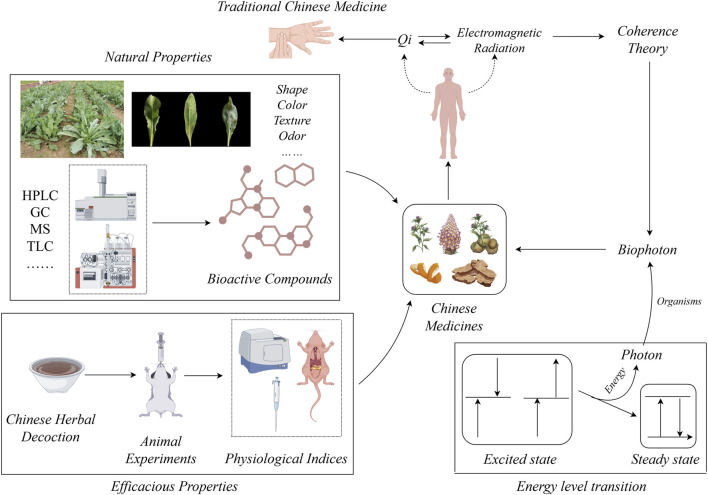
The association graph of Chinese herb-TCM-the human body-BE.

Finally, we summarize studies integrating biophoton technology with the quantification of medicinal properties, quality evaluation, and identification of processed medicinal products (Table 2). Based on the theoretical framework explored in this paper, we propose a visual model illustrating the relationships among TCM, medicinal plants, the human body, and BE (Figure 2). This figure provides a more intuitive display of the integration of theories and methods mentioned in this article. It further highlights the synergistic development of TCM and modern biophysics at both theoretical and practical levels. This model helps to understand the research ideas and specific paths of Chinese medicine, especially the medicinal properties of medicinal plants. Moreover, it provides an operational analytical framework for subsequent empirical research and the establishment of new systems, thereby promoting the in-depth development of interdisciplinary research.

**TABLE 2:** Application of BE detection technology in the areas of Chinese herbal research.

Application areas	Testing samples	Detection and analysis methods	Available indicators
Meridian tropism	Dried powder	DL (spectrum)	Peak wavelength
		SPE (spectrum)	​
Flavor	Dried powder	DL (bi-exponential decay parameter model)	y_0_, A_1_, T_1_, A_2_, T_2_
	Dried powder	DL (logistic regression)	N, S
Cold and hot medicinal properties	*Tetradesmus Obliquus* and herbal decoction	DL (Gu parameter model)	K
	Mice and herbal decoction	SPE (intensity)	SE ratio
Processing	Dried powder	DL (hyperbolic attenuation parameter model)	I_0_, Tau, Beta, T
		DL (bi-exponential decay parameter model)	y_0_, A_1_, T_1_, A_2_, T_2_
	*Tetradesmus Obliquus* and herbal decoction	DL (Gu parameter model)	K
Quality control	Dried powder	DL (bi-exponential decay parameter model)	y_0_, A_1_, T_1_, A_2_, T_2_
		DL (hyperbolic attenuation parameter model)	I_0_, Tau, Beta, T
	Fresh herbal samples	SPE (intensity)	CPS
		DL (Gu parameter model)	I_0_, T

### 6.3 Comparative analysis: biophoton technology vs. conventional quality assessment methods

Though BE technology shows promise, its position within the existing methods for medicinal herbal quality assessment must be clearly defined. Conventional methods, such as HPLC and its combination with mass spectrometry (LC-MS), form the cornerstone of modern quality control by providing precise, quantitative data on specific bioactive markers. Serum medicinal chemistry and pharmacodynamic studies further bridge the gap between chemical composition and internal therapeutic efficacy. However, as mentioned earlier, these approaches are essentially reductionist, focusing on discrete parts, whether components or efficacy, and often overlooking the holistic nature of medicinal herbs, a fundamental principle of TCM theory. Therefore, to objectively evaluate the complementary role of BE technology, we conducted a comparative analysis across several key dimensions, as summarized in Table 3. The primary advantages of BE detection lie in its non-destructive nature, rapid analysis (seconds to minutes), minimal sample requirement, and low operational cost after initial instrument investment. Most importantly, it provides a holistic fingerprint of the sample, capturing information related to the overall physiological, comprehensive components, and energetic state of the medicinal plant. This makes it more suitable for rapid identification of herbal properties and overall quality grading in a manner consistent with TCM holism. Conversely, conventional chromatographic methods well in precise quantification of specific chemical constituents, which is indispensable for standardizing known active ingredients and identifying toxic components. Their limitations include being time-consuming, destructive, requiring complex sample preparation (extraction, purification), and high costs for reagents, solvents, and reference standards. Crucially, they struggle to directly reflect the synergistic interactions of multiple components that constitute the holistic medicinal property. It is worth noting that BE technology should not be regarded as a replacement for established chemical assays but as a powerful complementary tool. However, there are currently no unified suppliers for BE instrumentation. Systems are predominantly assembled from components acquired via various channels, which causes a drawback of varying instrumental sensitivity and make results from different origins incomparable. Furthermore, mature methodological systems have yet to be established. Although these reflect the shortcomings of current BE detection technology, this also outlines the path forward for BE technology.

**TABLE 3:** Comparative analysis: BE technology vs. conventional quality assessment methods.

Dimension	BE Technology	Conventional methods (e.g., HPLC, serum medicinal chemistry)
Philosophical basis	Holism; aligns with TCM theory of “qi” and systemic properties	Reductionism; split the whole into parts
Measured target	Characteristic parameters of BE	Specific chemical compounds (markers, active ingredients)
Sample preparation	Minimal or none (direct measurement of some powder or only a fresh plant tissue, like a leave)	Extensive (drying, grinding, extraction, purification, and derivation from a sample of a certain weight, usually ranging from a few grams to tens of grams)
Detection speed	Very fast (seconds to minutes for a single measurement)	Slow (several days for the condition exploration and minutes to hours per sample for chromatographic run; at least half a month of animal experiment cycle)
Destructiveness	Non-destructive; sample can be reused for other analyses	Destructive; sample is consumed during extraction, administration, and analysis
Operational cost	Low (after initial instrument investment; even no consumables)	High (cost of solvents, columns, standards, experimental animals, and waste disposal)
Information depth	Macro-level, functional state; good at distinguishing overall properties (e.g., cold/hot), processing, origin, and quality	Micro-level, compositional; excellent for quantifying specific compound concentrations or identifying the substance basis of pharmacological effects
Quantification capability	Semi-quantitative; based on intensity, decay parameters, or K-value; high precision for holistic comparison	Highly quantitative; provides exact data with high accuracy and reproducibility
Key strength	Rapid, holistic identification; non-destructive; ideal for property identification and quality trend monitoring	Precise quantification of known compounds; essential for standardization and safety control
Key limitation	Cannot identify or quantify specific chemical constituents; results can be influenced by environmental factors; different sensitivities between instruments	Cannot directly reflect holistic properties; time-consuming and expensive for large-scale screening
Primary application	Quality grading, medicinal property determination, origin identification, processing discrimination, real-time monitoring	Quantification of active ingredients, toxic components, fingerprinting for authentication, serum medicinal chemistry and pharmacokinetic studies

## 7 Conclusion

Chinese herbs, as the cornerstone of TCM therapeutics, play critical roles in disease management and the maintenance of human health. The therapeutic potential of Chinese herbs is governed by their intrinsic “biases.” A comprehensive understanding of these properties must encompass both the efficacious properties (e.g., four qi, five flavors, meridian tropism), observed through the herbal interaction with the human body, and the natural properties, arising from the medicinal plant’s interaction with the environment during growth. Current pharmacological evaluation models, predominantly reductionist and focused on post-intervention organismic responses, inadequately capture the holistic and internal aspects of herbal quality. This limitation, coupled with a lack of TCM-aligned quality control indicators, contributes to inconsistent herbal quality and diminished clinical outcomes. The cultivation process, in particular, remains outside the quality control framework and is still a critical gap in the quality assurance framework. To address these challenges, this review has established a theoretical framework linking TCM theory and BE and then systematically outlines the applications of this technology in medicinal plant research. We reviewed the initial application of biophoton technology in the analysis of herbal decoction characterization and then summarized the research progress in the quantification of the medicinal properties, the identification of processed products, and quality evaluation. Especially during the cultivation process, this technology enables real-time monitoring of the intrinsic physiological and metabolic states of medicinal plants, demonstrating significant potential for achieving true quality “control.” As a life phenomenon that conveys holistic biological information, BE may ultimately reveal the quantum thermodynamic foundations of Chinese medicinal properties. This thinking mode enables dual-aspect evaluation, integrating efficacious signatures with natural properties, and ensures the effectiveness of medicinal plants while preserving the systematic philosophy of TCM. Such methodology represents an epistemological bridge between traditional herbal cognition and modern quantum biological validation.
